# *Emilia coccinae* (SIMS) G Extract improves memory impairment, cholinergic dysfunction, and oxidative stress damage in scopolamine-treated rats

**DOI:** 10.1186/s12906-015-0864-4

**Published:** 2015-09-23

**Authors:** Harquin Simplice Foyet, Hervé Hervé Ngatanko Abaïssou, Eglantine Wado, Emmanuel Asongalem Acha, Ciobica Alin

**Affiliations:** Department of Biological Sciences, Faculty of Science, University of Maroua, Cameroon. P.O. Box: 814, Maroua, Cameroon; Department of Life and Earth Sciences, Higher Teachers’ Training College, University of Maroua, P.O. Box: 55 Maroua, Cameroon; Department of Agriculture, Cattle farming and Derived products, High Institute of the Sahel, University of Maroua, P.O. Box: 46 Maroua, Cameroon; Department of Biomedical Science, Faculty of Health Sciences, University of Buea, P.O. Box 63 Buea, Cameroon; Alexandru Ioan Cuza University, 11 Carol I Blvd., 700506 Iasi, Romania; Center of Biomedical Research of the Romanian Academy, Iasi Branch, Romania

**Keywords:** *Emilia coccinae*, Scopolamine, Antioxidant, Acetylcholine, Spatial memory

## Abstract

**Background:**

*E. coccinae* (SIMS) G. (Asteraceae) is an annual plant commonly found throughout the plain of the Central Africa and widely used in Cameroonian folk medicine for the treatment of fever and convulsions in children. We previously reported that the methanolic extract of this plant improved spatial memory. However no underlying mechanism was explored. The present study was undertaken to investigate the effects of the hydroalcoholic extract of *Emilia coccinae* on memory in scopolamine treated rats and to propose possible mechanisms of action.

**Methods:**

Novel object recognition and Y-maze paradigm were used to test memory while oxidative profile, AChE and ACh level of the whole brain were assessed to outline the mechanism of nootropic activity of the extract. 200 and 400 mg/kg of the extract were chronically administrated during 14 consecutive days in separate groups of scopolamine intraperitoneal treated rats (1.5 mg/kg).

**Results:**

The hydroalcoholic extract of *Emilia coccinae* (HEEC) at the dose of 200 mg/kg significantly improved the memory of rats and reversed the amnesia induced by scopolamine. In addition, we showed that this extract is decreasing the acetyl cholinesterase activity while also increasing the acetylcholine levels in the brain. HEEC (200 and 400 mg/kg) significantly increased antioxidant enzyme activities (SOD, GSH and CAT) and reduced lipid peroxidation (MDA level) in the rat whole brain homogenates.

**Conclusions:**

Taken together, our results suggested that the hydroalcoholic extract of *Emilia coccinae* ameliorated the cognitive dysfunction in scopolamine treated rats through the blockage of the oxidative effect of scopolamine and inhibition of AChE activity.

## Background

Alzheimer’s disease (AD) is a neurodegenerative disease related to cognitive and behavioral impairments, characterized by loss or decline in memory and severe cognitive impairments.

Older age, genetic predisposition, other acquired medical conditions, stress and emotions are factors that may contribute to various deficiencies observed in AD such as memory loss, impaired learning, and dementia or to more ominous threats like Parkinson’s and Alzheimer’s diseases [1, 2].

To date, the main cause of AD remains unclear, but it is considered that the β-amyloid and tau protein aggregation, reduced acetylcholine (ACh), and glutamatergic deficit are regarded as principal pathogenesis of AD [3]. Recent studies have speculated that free radicals produced during oxidative stress and/or inflammatory processes are also pathologically important in AD [4]. Accumulation of free radical damage and alterations in the activities of antioxidant enzymes such as superoxide dismutase and catalase have been observed in the central nervous system of AD patients [5]. Moreover, in AD and mild cognitive impairment brains, the increased oxidative damage to lipids and proteins and the decline of glutathione and antioxidant enzyme activities correlate with the severity of the disease, suggesting that oxidative stress may be one of the alterations that occur during the initiation and development of AD [6, 7].

In the last decades many studies have been performed in order to develop an efficient drug for AD therapy. However, very few of them have obtained the final approvals in most countries.

Unfortunately, none of the drugs can reverse nor stop the course of the disease. In this way, most of these substances, which are used for the symptomatic treatment of AD since 1990, are cholinesterase inhibitors that prolong the acetylcholine (ACh) availability after it is released from cholinergic nerve endings, through the inhibition of acetylcholinesterase (AChE). However, non selectivity of these drugs, their limited efficacy, and poor bioavailability, adverse cholinergic side effects in the periphery, narrow therapeutic ranges, and hepatotoxicity are among the several limitations to their therapeutic success [8, 9].

Moreover, the drugs could exert several important side effects including diarrhea, nausea, insomnia, muscles cramps, vomiting, fatigue and loss of appetite [10]. Thus, considering the fact that the management of AD can be a major challenge for the health care systems, all around the globe many research are now undertaken in order to valorize phytopharmaceutical alternatives approaches which is widely available and accessible at low costs. In this way, numerous plants like *Areca catechu* [11], *Boswellia papyrifera* [12] or *Malva parviflora* [13], have been reported to treat cognitive disorders; learning and memory disorders, which are commonly observed in AD. Thus, there is a growing interest in the use of plant extract to improve leaning, memory and general cognitive function, with a large number of reviews highlighting the benefits of some phytochemicals as brain function modulators [14, 15]. Moreover, in one of our previous studies, we reported the *in vitro* antioxidant effects of *Emilia coccinae*, which could have been correlated also with some facilitating effects of the same extract on an animal model of anxiety and depression [16]. In that study, the standardized methanolic extract of *Emilia coccinae* significantly increased the number of open arm entries and time spent in the open arms of the elevated plus maze test and was as effective as Imipramine in inducing shortening of immobility time in forced swimming test.

Phytochemical analysis revealed that the dry leaves contained 863.04 ± 5.42 mg of GAE/100 g of dry material, which represents a very good content of total phenolics compounds while the total reducing power was about 4.71 ± 0.04 g of Vit C equivalent/100 g of dry material.

Some studies hypothesized that the presence of depression or anxiety would hinder some aspects of memory performance. Depression, when compounded by anxiety, has not only an adverse effect on immediate recall and amount of acquisition, but also on the retrieval of newly learned information [17], illustrating the effects of comorbidity on memory and learning. It thus appeared reasonable for us to explore the effect of this extract with both anxiolytic and antidepressant activities in short-term memory.

In fact, *E. coccinae* (SIMS) G. (Asteraceae) is an annual plant commonly found throughout the plain of the Central Africa and in dry area up to 2000 m altitude in the eastern Africa [18]. This species belongs to the genus Emilia represented by about 100 species, with 50 of them found in Africa [18]. In the folk African traditional medicine, this plant is used for the treatment of fever and convulsions in children [19]. The dry leaves are used for the treatment of wounds, sores and sinusitis ulcer, ringworm [20]. In addition, in some tribe in the western part of Cameroon, the infusion of the dry leaves of this plant is used as a potent sedative and restorative.

Therefore, considering the promising behavioral result obtained with this specie, the modest cognitive benefit offered by the available treatments with some time the severe side effects, we decided to investigate the effect of chronic administration of the hydroalcoholic extract of *E. coccinae* in a experimental model of cognitive impairment in rats, as induced by scopolamine, an anti-muscarinic drug.

## Methods

### Plant material and extraction

Fresh leaves of *E. coccinae* were harvested in February 2013 at Etoug Ebe in the Centre Region of Cameroon and authenticated at the National Herbarium-Yaoundé, where the voucher specimen was conserved under the reference number 6297/HNC. The leaves were washed and dried at room temperature (24–26 °C during 10 days) and pulverized into a coarse powder using a suitable grinder. The powder was stored in a dark and airtight container and kept in −30 °C until further analysis.

### Hydroalcoholic extraction

The preparation of the plant extract was as described by Haque et al. [21]. Briefly, 150 g of powdered material was placed in a clean, flat-bottomed glass container and soaked in 1.5 L of methanol/water (70/30, V/V). Then the extraction was carried out by using an Ultrasonic Sound Bath accompanied by sonication (45 °C, 25 min). The entire mixture underwent a coarse filtration through white cotton material. The extract obtained was filtered through Whitman filter paper (Bibby RE200, Sterilin Ltd., UK). The solution was then concentrated using a rotavapor in high vacuum up to 60 °C. The filtrate was later frozen and lyophilized to obtain the hydroalcoholic (27.90 g) extracts.

### Experimental animals

Male Wistar albino rats (n = 30), weighing 100–180 g at the beginning of the experiment, were obtained from the Laboratory of Phytopharmacology (LAPHYPHA) of the University of Dschang, Cameroun. The animals were housed in polyacrylic cages (6 animals/cage) and maintained in a temperature and light-controlled room (25 ± 2 °C, a 12-h cycle). The animals were acclimatized to laboratory condition for 7 days before the start of experiment. Prior to and after treatment, the animals were fasted for 12 and 7 h, respectively. However, all animals were allowed to drink water *ad libitum*. Rats were treated in accordance with the guidelines of the Cameroonian bioethics committee (reg N°.FWA-IRB00001954) and in accordance with NIH- Care and Use of Laboratory Animals manual (8th Edition). The present study was approved by the Ethic Committee of the Faculty of Sciences of the University of Maroua (Ref. N°14/0261/Uma/D/FS/VD-RC), Cameroon. Efforts were also made to minimize animal suffering and to reduce the number of animal used in the experiment. Each animal was tested in only one behavioral test. The experiments were performed in the morning (8–12 h), and the light level in the experimental room was 200 lux.

### Chemicals

Scopolamine, chloral hydrate, acetylthiocholine iodide, 5, 5-dithiobis (2-nitro-benzoic acid) (DTNB), 2-thiobabituric acid (TBA), Piracetam were purchased from Sigma–Aldrich, USA. All drugs and extracts were freshly prepared in saline on the day of the experiments. Scopolamine and piracetam was administered intraperitoneally (i.p.) to the rats while the extract was administered by gavage (per os, *p.o.*). Control animals received oral administration of 10 ml/kg body of the vehicle.

### Animal treatments

Rats were randomly allocated into five groups of 6 animals each as follows: Group I received saline solution and served as normal control. Group II received scopolamine (1.5 mg/kg) administered intraperitoneally and served as model group. Both groups received normal saline for 14 days. Groups III received piracetam (150 mg/kg) through intraperitoneal injection (i.p.). Group IV and V received respectively HEEC at the doses of 200 and 400 mg/kg, by gavage respectively for 14 days. The doses were fixed based on earlier studies on the hydroalcoholic extract of *Emilia coccinae* extract in our laboratory.

Scopolamine (1.5 mg/kg) as a disease inducer was administered as a single dose 30 min after drugs administration through intraperitoneal injection (i.p.) route to all the groups except normal control group. The same procedure was carried out on days 1, 4 and 8 because of the transient effect of the scopolamine on memory impairment [22, 23]. Behavioral testing was done on days 8 and day 14 following extract administration, 30 min after scopolamine injection. The aforementioned dosage and the duration of treatment were selected following a screening studies and previously published reports regarding *Emilia coccinae* biological effects [16] (see Table 1).

**Table 1:** Experimental design

Group	Treatment	Dose (mg/kg)
I	Normal control treated with saline solution	-
II	Negative control group treated with scopolamine	1.5 mg/kg, i.p
III	Piracetam + Scopolamine	150 mg/kg + 1.5 mg/kg, i.p
IV	Extract low dose + Scopolamine	200 mg/kg, p.o. + 1.5 mg/kg, i.p
V	Extract high dose + Scopolamine	400 mg/kg, p.o. + 1.5 mg/kg, i.p

### Behavioral evaluation

The animals’ behavioral activities were tracked and recorded using trial version of ANY-maze 4.9 behavioral software.

#### Y-Maze test

Y-maze analysis has been shown to be a reliable, noninvasive test to determine cognitive changes in Wistar rat through the measurement of the spontaneous alternation behavior in the Y-maze task [24].

The maze used in the present study consisted of three arms (35 cm long, 25 cm high and 10 cm wide) and an equilateral triangular central area.

All animals were tested in a randomized order at the start and end of the experimental protocol. Rats were treated once daily whit the hydroalcoholic extract of *E. coccinae* leaves (200 and 400 mg/kg, per os), piracetam (150 mg/kg, i.p.), or saline (10 ml/kg; per os) during 14 consecutive days. Thirty minutes after the aforementioned drug had been administrated; rats were placed at the end of one arm and allowed to move freely through the maze for 8 min. The time limit in Y-maze test was 8 min, and every session was stopped after 8 min. An arm entry was counted when the hind paws of the rat were completely within the arm. Spontaneous alternation behavior was defined as three consecutive entries in three different arms (i.e. A, B, C or B, C, A, etc.). The percentage alternation score was calculated using the following formula: Total alternation number/Total number of entries minus 2) x 100. Furthermore, the total number of arm entries was used as a measure of general activity of the animals. The maze was wiped clean with 70 % ethanol between each animal to minimize odor cues [25, 26].

#### Object recognition test

This test was performed as described by El-Marasy et al. [27]. Briefly, three days before testing, each rat was allowed to explore the apparatus for 2 min, while on the testing day, 30 min following scopolamine injection, a session of two trials, 2-min each was allowed. In the “sample” trial (T1), two identical objects were placed in two opposite corners of the apparatus. A rat was placed in the apparatus and was left to explore these two identical objects. After T1, the rat was placed back in its home cage and an inter-trial interval of 1 h was given. Subsequently, the “choice” trial (T2) was performed. In T2, a new object (N) replaced one of the objects that were presented in T1, then rats were exposed again to two different objects: the familiar (F) and the new one (N). Exploration was defined as follows: directing the nose toward the object at a distance of no more than 2 cm and/or touching the object with the nose. From this measure, a series of variables were then calculated: the total time spent in exploring the two identical objects in T1, and that spent in exploring the two different objects, F and N in T2.

The distinction between F and N in T2 was measured by comparing the time spent in exploring the F with that spent in exploring the N. DI is the dissimilarity index and represents the difference in exploration time expressed as a proportion of the total time spent exploring the two objects in T2.

DI was then calculated using the following formula:

$$ DI=\frac{N-F}{N+F} $$

### Estimation of biochemical parameters

#### Brain tissue preparation

The rats were decapitated on day 14^th^ after the last behavioral testing under chloroform anesthesia. The skull was cut open and the brain was exposed from its dorsal side. The whole brain was immediately removed and cleaned with chilled normal saline on the ice. A 10 % (w/v) homogenate of brain samples (0.03 M sodium phosphate buffer, pH 7.4) was prepared. The homogenate was centrifuged (15 min at 3000 rpm) and the supernatant was used for assays of SOD, CAT activities, total GSH content and MDA level. In addition, the levels of AChE and ACh were estimated.

#### Estimation of antioxidant enzymes

##### Determination of SOD

The activity of superoxide dismutase (SOD) was assayed by monitoring its ability to inhibit the photochemical reduction of nitroblue tetrazolium (NBT). Each 1.5 mL reaction mixture contained 100 mM Tris/HCl (pH 7.8), 75 mM NBT, 2 M riboflavin, 6 mM EDTA, and 200 L of supernatant. Monitoring the increase in absorbance at 560 nm followed the production of blue formazan. One unit of SOD is defined as the quantity required to inhibit the rate of NBT reduction by 50 % as described by Winterbourn et al. [28].

##### Estimation of GSH

To measure the reduced glutathione (GSH) level, the tissue homogenate (in 0.1 M phosphate buffer pH 7.4) was taken. The procedure was followed as previously described by Shamnas et al. [29]. Briefly, the homogenate was added with equal volume of trichloroacetic acid (TBA, 20 %) containing 1 mM EDTA to precipitate the proteins. The mixture was kept for 5 min prior to centrifugation. The supernatant (200 μl) was then transferred to a new set of test tubes and added 1.8 ml of the Ellman's reagent (5, 5'-dithio *bis-*2-nitrobenzoic acid) (0.1 mM) was prepared in 0.3 M phosphate buffer with 1 % of sodium citrate solution). Then all the test tubes rose to the volume of 2 mL. After the completion of the total reaction, the solutions were measured at 412 nm against blank. Absorbance values were compared with a standard curve generated from standard curve from known GSH.

##### Determination of CAT

Catalase (CAT) activity was assayed following the method of Hritcu et al. [30]. The reaction mixture consisted of 150 μL phosphate buffer (0.01 M, pH 7.0), 100 μL supernatant. Reaction was started by adding 250 μL H_2_O_2_ 0.16 M, incubated at 37 °C for 1 min and reaction was stopped by the addition of 1.0 mL of dichromate acetic acid reagent. The tubes were immediately kept in a boiling water bath for 15 min while the green color developed during the reaction and was read at 570 nm on a spectrophotometer. Control tubes, devoid of enzyme, were also processed in parallel. The difference in absorbance per unit was used as the measure of catalase activity.

##### Determination of MDA

Malondialdehyde (MDA), which is a measure of lipid peroxidation, was spectrophotometrically measured using the thiobarbituric acid assay [7, 31]. 200 μL of supernatant added and briefly mixed with 1 mL of 50 % trichloroacetic acid in 0.1 M HCl and 1 mL of 26 mM thiobarbituric acid. After the vortex mixing, samples were maintained at 95 °C for 20 min. Furthermore, samples were centrifuged at 3000 rpm for 10 min and supernatants were read at 532 nm. A calibration curve was constructed using MDA as standard and the results were expressed as nmol/g protein.

##### Estimation of brain neurotransmitter

Estimation of acetylcholinesterase (AChE) and Acetylcholine (Ach) activities

Acetylcholinesterase activity was estimated by using an artificial substrate, acetylthiocholine (ATC). In the medium, thiocholine released due to the cleavage of ATC by AChE is allowed to react with the -SH reagent 5,5’-dithiobis-2-nitrobenzoic acid (DTNB), which is reduced to a yellow colored anion called thionitrobenzoic acid, measurable at the wave length 412 nm. Concentration of thionitrobenzoic acid was spectrophotometrically detected and taken as a direct estimate of the AChE activity [32]. Acetylcholine level in the whole brain was estimated using Hydroxylamine method as described by Stepankova et al. [33]. The reaction mixture was prepared and mixed with the aqueous hydroxylamine hydrochloride and 3.5 M aqueous KOH (1:1 v/v). The resulting mixture was mixed 2 min. to convert ACh totally to acethydroxamic acid. The pH value was changed again by adding Conc. HCl/H2O (1:2 v/v). The reddish brown color formed after adding 0.37 M ferric nitrate was read at 540 nm. The Ach content was calculated with reference to the standard values.

### Statistical analysis

Data was presented as mean ± SEM. One-way ANOVA (for Y-maze data) and two-way ANOVA (for novel object recognition and Y-maze data followed by Bonferoni Multiple Comparison Test was performed using Graph Pad Prism version 5.00 for Windows, Graph Pad Software, San Diego California USA. A probability level of 0.05 or less was accepted as significant. Pearson’s correlation coefficient and regression analysis were used to evaluate the connection between behavioral responses and biochemical parameters.

## Results

### *Effects of the extract in the* Y-Maze task

**Fig. 1:**
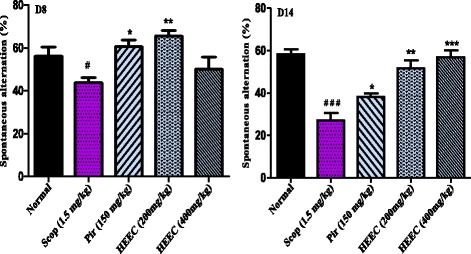
Effect of the hydroalcoholic extract of *E. coccinae* leaves and piracetam (Pir) on the spontaneous alternation percentage after 8 and 14 days of treatments on scopolamine (Scop) treated animal in Y-maze task. Each column represents mean ± S.E.M. of 6 animals. Data analysis was performed using One way ANOVA followed by Bonferroni posttests. *P < 0.05; **P < 0.001; **P < 0.0001 vs. control animals; ^#^ *P* < 0.05 vs. normal animals

**Fig. 2:**
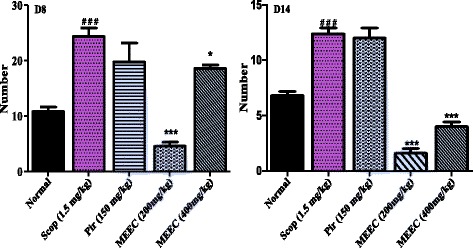
Effect of the hydroalcoholic extract of *E. coccinae* leaves and piracetam (Pir) on the locomotory activity of animals in Y-maze task. Each column represents mean ± S.E.M. of 6 animals. Data analysis was performed using One way ANOVA followed by Bonferroni posttests. *P < 0.05; **P < 0.001; **P < 0.0001 vs. control animals; ^#^ *P* < 0.05 vs. normal animals

In Y-maze task, the hydroalcoholic extract of *E. coccinae* leaves significantly increased the spontaneous alternation behavior in rats with cognitive deficit-induced by scopolamine after eight days administration compared to the control group, (F (4, 12) = 3.25, *P* < 0.0102) (Fig. 1). Daily oral administration of HEEC (200 and 400 mg/kg) for 14 consecutive days before scopolamine injection also significantly increased the correct trials of rat in the Y-maze compare to scopolamine non treated group suggesting effects on short term-memory. Post-Hoc analysis revealed no significant difference between the 2 groups of extract. Also, post hoc analyses revealed significant statistical differences (*p* < 0.0001) between animals exposed to scopolamine and those treated with HEEC (Fig. 1). Likewise, the HEEC (200 and 400 mg/kg) substantially reduced the number of line crossed in the Y-maze (Fig. 2). However, this inhibition of the locomotory activity was more pronounced at the dose of 200 mg/kg and after 14 days of treatment (*p* < 0.0001) in comparison with the scopolamine-treated group. No significant reduction in the ambulatory activity was noted with piracetam.

### *Effects of the extract in the* Novel object recognition

**Fig. 3:**
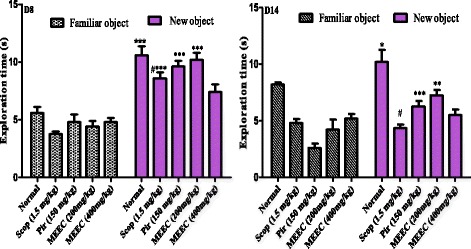
Figure 3 Effect of MEEC and piracetam (Pir) on the exploration time of the familiar vs. the novel object in the object recognition test. Rats received 14 daily administration of HEEC (200 mg/kg, p.o.), or donepezil (1.5 mg/kg, p.o.) used as a standard drug

**Fig. 4:**
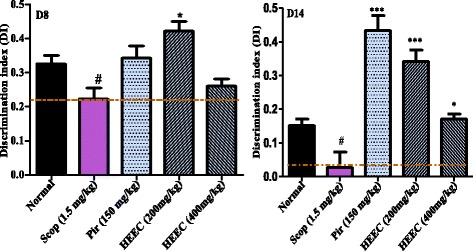
Effect of HEEC discrimination index (DI) of scopolamine-induced memory impairment in rats in the object recognition test. Rats received 14 daily administration of HEEC (200 and 400 mg/kg, p.o.) or Piracetam (150 mg/kg, i.p.) used as a standard drug

All rats treated with the hydroalcoholic extract from leaves and piracetam, as well as the control animals took more time to explore new object after 8 or 14 days of treatment. However, the time taken by the animals subjected to scopolamine injection was significantly less than that of normal animals, indicating the impairment of the memory process. The treatment of the animals by Piracetam and HEEC (200 mg/kg) significantly ameliorated the exploratory time of the rats in this task as compared to scopolamine group (Fig. 3). Although the exploratory time increased at the dose of 400 mg/kg, this was not significant when compared to scopolamine group. ANOVA of all differences yielded F (2, 38) = 4.034; (*p* < 0.032). Discrimination Index data revealed a significant effect of HEEC after 8 days (F (4, 18) = 2.92, *P* < 0.0015) and after 14 days (F (3, 8) = 4.06, *P* < 0.0003) of treatment. Post hoc comparisons indicated that the HEEC 200 and 400 mg/kg-treated rats discriminated significantly better *N* than *F* with respect to their scopolamine-treated counterparts (*P* < 0.05, 8 days; and *P* < 0.001, 14 days) (Fig. 4).

### Effect of the hydroalcoholic extract of *E. coccinae* on brain lipid peroxidation and antioxidant enzymes

#### Measurement of lipid peroxidation

**Fig. 5:**
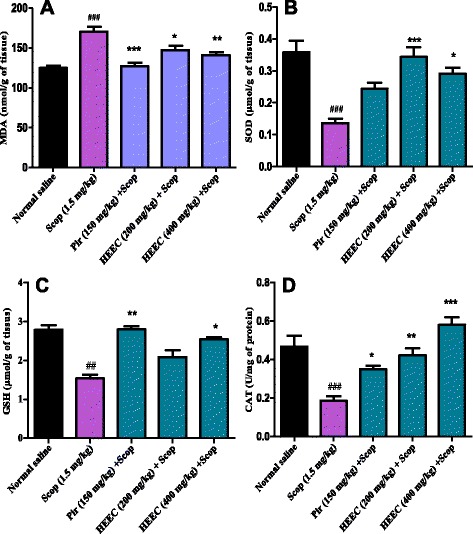
Effect of the hydroalcoholic extract of *E. coccinae* administration on MAD (**a**) SOD (**b**), GSH (**c**) and CAT (C) level of the brain tissue. Data was analyzed by one-way ANOVA followed by Bonferroni post-hoc test. Each column represents mean ± S.E.M. of 6 animals. ***p < 0.001, **p < 0.01 and *p < 0.05 when compared to scopolamine treated group; ^###^ *p* < 0.001, when compared to normal animals

As shown in Fig. 5a, pre-treatment with hydroalcoholic extract of *E. coccinae* (200 and 400 mg/kg) for 14 successive days shows in a dose dependant manner significantly reduction (F (3, 16) = 3.23, *p* < 0.00034) of the MDA level in respect to the scopolamine treated group. Post hoc analysis also showed a significant difference between scopolamine versus HEEC treated group (*p* < 0.05). However the decrease in MDA level of the piracetam and HEEC-treated group were still below that of normal rats.

#### Effect of HEEC on Superoxide dismutase

When treated with scopolamine, the level of brain SOD decreased significantly (*p* < 0.001) when compared with that of normal animals. One way ANOVA followed by post hoc test showed that the treatment of amnesic rats with HEEC (200 and 400 mg/kg) significantly (F (4, 20) = 2.86, *p* < 0.00023) increased SOD level when compared to scopolamine group (Fig. 5b). The standard drug piracetam (150 mg/kg) also increased the SOD level but this was not significant.

#### GSH activity in the brain

The effect of hydroalcoholic extract of HEEC on glutathione content in the brain is summarized in Fig. 5c. The GSH level of brain homogenate in scopolamine group (1.54 ± 0.27) was found to be significantly lower (*p* < 0.01) than the GSH level in normal group (2.78 ± 0.21). At the end of the treatment period with HEEC (200 and 400 mg/kg) or piracetam (150 mg/kg), GSH level was found to be increased in a highly significant manner (*P* < 0.05, 400 mg/kg; *P* < 0.001, piracetam). Piracetam almost completely restored the glutathione level in scopolamine treated groups to the normal level.

#### CAT activity in the brain

As shown in Fig. 5d, scopolamine injection to the rats brings to highly significant reduction (*p* < 0.001) in CAT level in the rats brain. The treatment of the scopolamine injected rats with the plant extract resulted in a significant and dose dependent increase in catalase activity when compared with Scopolamine injected rats.

**Fig. 6:**
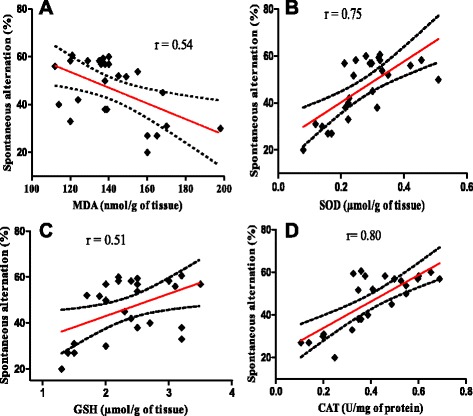
Correlation between spontaneous alternation percentage vs. MDA (**a**), spontaneous alternation percentage vs. SOD (**b**), spontaneous alternation percentage vs. GSH (**c**) and spontaneous alternation percentage vs. CAT (**d**) in the hydroalcoholic extract-treated groups (200 mg/kg); with the 95 % confidence band of the best-fit line

More importantly, a significant positive correlation was evidenced by determination of the linear regression between SOD vs. spontaneous alternation (r = 0.75, *p* < 0.0002) (Fig. 6a) and CAT vs. spontaneous alternation (r = 0.80, *p* < 0.0002) (Fig. 6d) in scopolamine exposed groups treated with the hydroalcoholic extract of *E. coccinae* (200 mg/kg). The correlation between GSH vs. spontaneous alternation was positive but very weak (r = 0.51, *p =* 0.017) (Fig. 6c) while significant negative correlation was obtained between MDA and spontaneous alternation percentage (r = 0.54, *p =* 0.0073) (Fig. 6a).

### Effect of hydroalcoholic extracts of *E. coccinae* on brain AChE and Ach activities

**Fig. 7:**
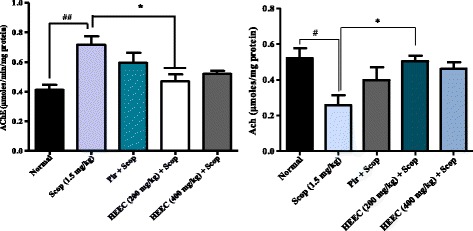
The effect of 14 days oral administration of the hydroalcoholic extract of *E. coccinae* (HEEC, 200 and 400 mg/kg) and piracetam (Pir, 150 mg/kg) on AChE and Ach activity in rat’s brain

The effect of hydroalcoholic extracts of *E. coccinae* in brain AChE and Ach activities are shown in Fig. 5. Scopolamine administration resulted in significant increase in the AChE activity with respect to Normal group). Meanwhile, hydroalcoholic extracts of *E. coccinae* significantly (F (2, 86) = 6.04, *p* < 0.0023) reduced AChE activity reaching similar values to normal group (Fig. 6). These results show that administration of HEEC suppressed the increase of AChE activity by scopolamine administration. At the same time, was notice a significant decrease in the Ach content in the brain of scopolamine treated rats (Fig. 7). One way ANOVA analysis revealed that in contrast to the Ach reduction, the rats treated with HEEC and piracetam shows a significant recovery (F (2, 86) = 4.22, *p* < 0.012) on Ach content in the brain region. However with the Bonferroni's Multiple Comparison post Test, this recovery was significant (*P* <0.05) only with the HEEC at the dose of 200 mg/kg.

## Discussion

In this report, we demonstrate that sub chronic treatment with HEEC improved learning and memory behaviors in adult male rats subject to scopolamine memory disruption. Specifically, we have shown that the oral administration of HEEC (200 and 400 mg/kg) after 8 and 14 days ameliorated the memory retention in the novel object recognition task and improved spatial learning in the Y-maze. Acetylcholine is a neurotransmitter acting on cholinergic receptors and widely distributed throughout the brain. During the last decade, it has long received much attention in memory research among neuroscientist. It is well established that alteration in the levels of acetylcholine or AChE activity may affect the cholinergic transmission process and leads to learning and memory a deficit which imitates Alzheimer’s disease [34]. Scopolamine, a nonselective muscarinic antagonist block cholinergic signaling and produce memory and cognitive dysfunctions, causing learning and memory deficits including long-term and short term memory impairment [35]. Consequently, intraperitoneal administration of the muscarinic antagonist scopolamine was widely and successfully exploited as a pharmacological model for AD in rats. Scopolamine induces dysregulation of the cholinergic neuronal pathway and memory circuits in the central nervous system, resulting in serious impairments in learning, acquisition, and short-term retention of spatial memory tasks [36].

In the present study we used two well-characterized memory tasks: Y-maze and novel object recognition to evaluate the effect of the HEEC on the scopolamine model of AD in rats. Spontaneous alternation behavior determined using the Y-maze test has been viewed as an indicator of spatial short-term memory [30]. In this test, rats must remember the arm most recently entered in order to alternate arm choice. Furthermore, treatment with scopolamine has been demonstrated to impair spontaneous alternation behavior in animal models [37]. In the present study, spontaneous alternation behavior in scopolamine treated rats was significantly lower than in rats treated with vehicle. In contrast, HEEC significantly reversed the cognitive deficit induced by scopolamine in the Y-maze task. Together, these results suggest that that HEEC enhance short term or working memory. In the same behavioral task, in contrast with piracetam, the hydroalcoholic extract of *E. coccinae*, especially at the dose of 400 mg/kg, significantly decreased the number of lines crossed by the rats (ambulatory activity). It is somehow difficult to evaluate the motor activity of the extract with some variety of locomotory manifestations. In our previous experiment using the same extract at the same doses [16], the motor activity (number of lines crossed) in the open field was decreased with an increase of the time spent at the center of the maze as compared to control. At the same time on the EPM platform, locomotor index- number of entrance in the closed arms decreased in the extract, as compared to control. The correlation of anxiety and memory parameters could thus be relevant since rats were displayed lower anxiety when treated with HEEC (200 and 400 mg/kg). So the increased activity in Y-maze may be mainly due to memory facilitation by the HEEC, resulting in increased searching in the maze and the better spatial memory that we observed.

The novel object recognition test measures the natural propensity of a rat to explore a novel versus familiar object. Our results showed that exploration of the novel object was reduced in animals exposed to intraperitoneal injection with scopolamine. This task has both an exploratory behavior component as well as a memory retention component such that an animal must have sufficiently explored the familiar object during the pretest phase in order to distinguish between it and a novel object later during the test phase [38]. In this study, scopolamine-treated animals exhibited less total exploration time during pre-training than normal animals. This is similar to previous findings in other behavioral paradigms showing that scopolamine-treated adult animals exhibit decreased exploratory behavior and significantly reduction in the discrimination index in the NOR task. After the pre-treatment by HEEC, two-way analysis of variance followed by Bonferroni post test revealed a significant effect of the treatment on the total amount of time spent exploring the novel objects as well as on the discrimination index compare to scopolamine group. This indicate that the extract sustain memory formation in rats injected with scopolamine-induced dementia rat model.

Cholinergic transmission generally ends by the acetylcholine hydrolysis through the enzyme AChE which is responsible for degradation of Acetylcholine to acetate and choline at the level of the synaptic cleft. In this way, AD has also been correlated with the loss of cholinergic neurons and decreases in the levels of acetylcholine (ACh) and choline acetyltransferase (ChAT) [39]. Lesions in these pathways result in decreased Ach release and thus cause learning and memory dysfunction. Until now, inhibition of acetylcholinesterase (AChE) has served as a strategy for the treatment of AD, senile dementia, ataxia, and Parkinson’s disease [40, 41].

Thus, estimating the acetylcholine esterase activity can provide valuable information on cholinergic function, which can correlate with cognitive function. In that way, AChE inhibitors play an important role in the nervous system disorders, owing to their potential as pharmacological and toxicological agents. Recently, some AChE inhibitors like tacrine and rivastigmine were used in the treatment of Alzheimer’s disease [9].

Our results clearly indicated that the administration of scopolamine to experimental animals was followed by a significant increase in the AChE activity in the brain, especially in normal rats. The administration of the plant extract for two weeks decreased acetylcholine esterase activity in brain homogenate of rat subject to scopolamine memory impairment. As a result, there was an increase of Ach Expression in brain hence triggering of cholinergic firings. This may be one of the mechanisms explaining the ability of the HEEC to enhance memory and cognition in this study. In present study, HEEC significantly decreased the AChE levels in the rat whole brain homogenate, indicating its potential in the attenuations of severity of AD. However, an absolute conclusion can’t be made on the AChE inhibitory effect of this extract since the effect of extract alone on AChE activity was not measured in this study.

In the scopolamine-induced AD animal model, the well-replicated amnesic effect of scopolamine has been identified as a principal consequence of the blockade of post-synaptic muscarinic M1 transmission, leading to disruption in the functioning of the hippocampus in working memory [36]. Other suggested mechanism is the increased oxidative stress in brains by scopolamine, inducing the activation of a cascade of redox-sensitive cell signal pathways [42]. Brain cells are known to contain a very high percentage of long chain polyunsaturated fatty acids. ROS are continuously generated in the nervous system during normal metabolism and normal neural activity. The brain is regularly subject to free radical-induced lipid peroxidation. It is also known that the protective system in the brain is poor against oxidative stress, compared to other tissues [43]. In this way, the estimation of the lipid peroxidation end product is commonly used as the biomarkers for *in vivo* lipid peroxidation in neurodegeneration research [44].

The results of antioxidant study showed decreased levels of SOD, CAT, and GSH in the scopolamine-induced dementia rat compared to normal rats. The protective system of the organism against reactive oxygen species is very complex, including enzymatic and non enzymatic biological process. These include SOD, that catalyses the conversion of superoxide radicals to hydrogen peroxide, which is then converted into water by GPX and catalase [45].

After 8 and 14 days pretreatment of the scopolamine-induced dementia rat with the hydroalcoholic extract of *E. coccinae* (200 and 400 mg.kg), there was a significant improvement of the levels of SOD, CAT and GSH whereas MDA level was decreased significantly. These results suggest that the plant extract has an *in vivo* antioxidant activity and is capable of ameliorating the effect of ROS in the brain of rats.

In a previous study, we reported that the methanolic extract of *E. coccinae* asper leaves improved spatial memory in rat through a mechanism that involved antioxidant and neuroprotective activities. Furthermore, we observed that this extract was able to reduce iron (Fe^3+)^ into Fe^2+^ using and scavenged 2, 4-dinitrophenyl-1-picryl hydrazyl (DPPH) radical *in vitro* [16]. This give us more confidence to conclude that the attenuation of the scopolamine neurotoxicity by the hydroalcoholic extract of *E. coccinae* in the present study may be due to the antioxidant propensity of this extract in the brain tissue. Despite these interesting results of the neuroprotective activity of HEEC, limitations of this work are that the AChE activity of the extract and histopathological studies of frontal lobes and hippocampus were not measured. This will be the main goal of our upcoming investigations.

## Conclusion

The present study demonstrates the beneficial effect of the hydroalcoholic extract of *E. coccinae* on scopolamine-induced cognitive impairment. In this way, *E. coccinae* protected from memory deficiency induced by scopolamine, as shown by behavioral tests using the Y-maze and Novel object recognition tests. Also, based on the results of the biochemical studies, it can be concluded that *E. coccinae* is an antioxidant; suggesting that it is possible the protective effects were related to its antioxidant effects. Although our results indicate that extract can ameliorate scopolamine induced AChE activity increment, we cannot say with great confidence that the extract has inhibitory effects on the enzyme because the effect of extract alone on AChE activity was not measured. Moreover, a significant positive correlation between SOD vs. spontaneous alternation and between CAT vs. spontaneous alternation was found when linear regression was performed. However, supplementary pharmacological and phytochemical investigations are necessary to clarify the molecular mechanisms of neuroprotection for these herbal extracts, as well as the principal bio-ingredient.
